# Effect of Core Density on the Three-Point Bending Performance of Aluminum Foam Sandwich Panels

**DOI:** 10.3390/ma16227091

**Published:** 2023-11-09

**Authors:** Peng Huang, Qiang Gao, Xixi Su, Zhanhao Feng, Xi Sun, Guoyin Zu

**Affiliations:** School of Materials Science and Engineering, Northeastern University, Shenyang 110819, China; neuhuangpeng@163.com (P.H.); 2110253@stu.neu.edu.cn (Q.G.); sux9573@gmail.com (X.S.); zhfengsincerity@gmail.com (Z.F.); sunxi0524@163.com (X.S.)

**Keywords:** aluminum foam, powder metallurgy, three-point bending test, energy absorption

## Abstract

Using the powder-metallurgy rolling method, aluminum foam sandwich (AFS) panels with a metallurgical bond between the foam core and the panel can be produced. In this study, by manipulating the foaming temperature and duration, AFS panels were fabricated with varying core densities and thicknesses, all maintaining a panel thickness close to 1 mm. Through the three-point bending test, this research deeply delved into how core density influences the mechanical behaviors of these AFS panels. It became evident that a rise in core density positively affects the bending strength and failure load of the panels but inversely impacts their total energy absorption efficiency. Differing core densities brought about distinct failure patterns: low-density samples primarily showed panel indentation and core shear failures, whereas those of high density demonstrated panel yield and fractures. Furthermore, the research offers predictions on the initial failure loads for different failure modes and introduces a comprehensively designed failure diagram, laying a foundational theory for the production of AFS panels.

## 1. Introduction

Aluminum foam is gaining attention due to its low density, high specific strength, and favorable acoustic and electromagnetic shielding properties [1,2]. By integrating metal panels on either side of this foam, one can fabricate aluminum foam sandwich (AFS) panels. This enhancement boosts not only the panel’s flexural strength but also its rigidity [2,3]. Such attributes have paved the way for its extensive application across industries, notably in the automotive, rail transportation, and aerospace sectors [4,5,6].

There are two primary methods to bond metal panels to aluminum foam: adhesive bonding and metallurgical bonding. The adhesive bonding technique predominantly uses epoxy resins and various adhesives to establish this bond, presenting a streamlined preparation process with highly tunable material properties. Metallurgical bonding employs methods such as welding [7,8,9], roll bonding [10,11,12], or extrusion bonding [13] to combine the panels with foam cores. Earlier investigations have predominantly concentrated on the influence of welding methods [8] and the employment of varied welding agents [9] on the sandwich panel’s efficacy. Metallurgical and adhesive bonding AFS show different failure modes during the three-point bending tests, and metallurgical bonding AFS shows better bending resistance and energy absorption properties [10,13]. Moreover, the bonding interface of adhesive bonding AFS is prone to failure at high temperatures, causing its mechanical properties to significantly decrease [14].

Historically, the quasi-static three-point bending test has been used to assess the mechanical performance of AFS panels. The observed failure modes include (a) indentation [10,14,15,16,17], (b) core shear [15,16,17], (c) face yield [14,15,16,17], (d) face wrinkling [18,19], and (e) face delamination [9,10,16,18,20,21]. Bart-Smith et al. [22] investigated the effects of panel strength on failure modes, indicating that specimens made from high-strength panels (6061-T6, 268 MPa) exhibited only indentation and core shear. In contrast, when using softer materials (6061-0, 80 MPa), face yielding was observed. Kabir et al. [23] noted that the AFS panel composed of low-strength face sheets (79.8 MPa, 0.32 mm thick), failure resulted in indentation and face yielding; however, the AFS panel with high-strength face sheets (262 MPa, 0.32 mm thick) exhibited both indentation and core yielding. Some researchers [17,24,25] investigated the effects of different face sheet and core layer thicknesses on failure modes. Their findings suggested that samples with thicker core layers were more prone to panel indentation [24,25]. Thin-skin specimens undergoing core shear (mode A) failure developed plastic hinges at the supports, while no plastic hinges were observed in thick-skin specimens experiencing core shear (mode B) [24]. Furthermore, thin-skin samples were more susceptible to face yielding compared to their thicker counterparts [17,25]. Some studies have shown that when the upper and lower face sheets vary in thickness [15,19] or when they are composed of different materials [16], multiple core shear failure modes can observe. Increasing the number of layers of carbon fiber can increase the flexural strength of the material [26]. Pandey et al. [21,27] investigated the influence of integrating carbon fibers at the interface on failure modes, finding that carbon fibers significantly enhanced the mechanical performance of AFS [27]. The addition of carbon fibers helped to prevent face delamination, enhancing the energy absorption efficiency of AFS [21]. Wang et al. [18] investigated the effect of adding glass fibers at the bonding interface and found that it prevented the bottom face sheet from fracturing. Yan, Chang et al. [20] examined the impact of using various epoxy resins on failure modes, revealing that the inclusion of acetone in epoxy resins helped avoid face delamination. Despite extensive scholarly research on the failure modes of AFS, the influence of core density on the performance of AFS has been less explored.

This study employs the powder-metallurgy rolling method to fabricate metallurgically bonded AFS panels. By adjusting the mold cavity height and powder volume, sandwich panels with varying core densities and thicknesses were fabricated. The compression experiments were conducted to determine the yield strength of foam core layers with varying densities. Based on these results, predictions were made regarding the influence of core layer density on the three-point bending performance of AFS. Through a comparative analysis of failure modes across core densities, we deduced the theoretical failure load values and subsequently constructed a comprehensive failure mode map, serving as a guide for practical manufacturing processes.

## 2. Materials and Experimental Procedures

### 2.1. Materials and Sample Preparation

In this research, the powder-metallurgy rolling method was used to prepare aluminum foam sandwich (AFS) panels with different core layer densities and thicknesses. The metal powders and TiH_2_ powder are mixed evenly in the mixer. The powder ratio and particle size are shown in Table 1 [10]. Adding Si, Mg, and Cu to Al can reduce the liquidus temperature of the preform, improving both the pore structure and maximum expansion [28]. The expansion rate of the preform composed of AlSi6Mg4Cu4 at 600 °C is 550% [29]. This high expansion rate makes the height of the foam aluminum sandwich panel easier to control. The top and bottom panels were made of 3003 aluminum alloy plates with dimensions of 500 mm × 350 mm × 4 mm. Along the length, aluminum strips securely weld the sides of these panels. While one end widthwise is fastened with rivets, the opposite end is similarly sealed, but only after it is loaded with the metal powder. By adjusting the width of the aluminum strips, casings with different cavity thicknesses are prepared. Specifically, two aluminum alloy casings were used in our experiments: (a) 500 mm × 350 mm × 28 mm with a 20 mm cavity height; (b) 500 mm × 350 mm × 23 mm, featuring a 15 mm cavity height.

**Figure 1:**
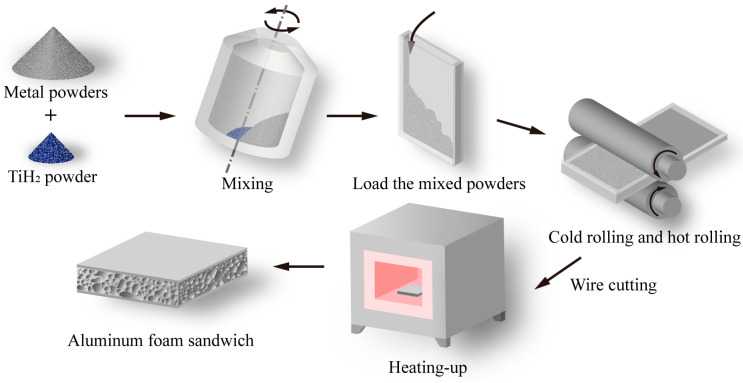
Schematic illustration of the preparation process for aluminum foam sandwich panels.

Utilizing two distinct casings filled with powder, we conducted successive cold and hot rolling processes, achieving an approximate reduction rate of 80%. This led to the creation of preforms with overall thicknesses of 6 mm and 4 mm. These preforms, after being resized to 200 mm × 200 mm through wire cutting, were subsequently positioned in a sintering mold and foamed at temperatures of 620 °C and 650 °C. The cavity size of the sintering mold was 250 mm × 250 mm, and its height was 15mm or 20mm. The heating rate of the preform in the sintering mold was approximately 50 °C/min. The liquidus temperature of AlSi6Mg4Cu4 is 596 °C [28], so the foaming temperature should be greater than this value. According to our previous research [29], we selected foaming temperatures of 620 °C and 650 °C and foaming times of 12 min and 16 min to prepare aluminum foam sandwich panels with different core layer densities. Post foaming, the AFS panels were edge-trimmed using wire cutting, thereby producing samples characterized by varied core densities and thicknesses. Figure 1 illustrates the aluminum foam sandwich panels’ fabrication process, while Table 2 delineates the specific preparation parameters.

### 2.2. Micro-Indentation Hardness Tests

**Figure 2:**
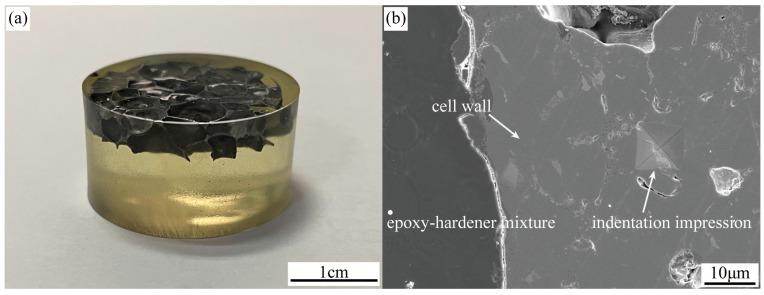
(**a**) Aluminum foam after inlay; (**b**) Indentation impression after a Vickers micro-indentation test on a foam cell wall.

To determine the yield strength of the core material, micro-hardness tests were performed on foam cores with varied densities utilizing the MH-5L digital micro-hardness tester. Initially, wire cutting was employed to detach the top and bottom panels from the AFS. The foam core was then resized into cylindrical samples measuring Φ20 mm × 5 mm. After these samples were cleaned and dried, they were positioned in a mounting mold. An epoxy resin-hardener (XJS-030, Shenyang Boyan Scientific Instrument Technology Co., Ltd., Shenyang, China) mix, in a 5:4 ratio, was poured into the mold and allowed to set for approximately an hour. To guarantee that the mounting material thoroughly penetrated the pores, the mounted mold was set inside a vacuum chamber. It is worth noting that the thickness direction of the sample contained minimal pores (1–2), ensuring complete pore filling during the inlaying process (as show in Figure 2a). This ensured that the mounting material offered adequate lateral support to the foam walls during micro-indentation. Finally, the mounted sample surfaces were refined using 800, 1500, and 3000 grit sandpapers.

The hardness tests were conducted on 30 samples at room temperature. For accuracy, the testing point on the foam wall was strategically chosen, ensuring that it was situated at a distance of at least three times the indentation’s diagonal length from the edge. In this test, a load of 200 g was applied and maintained for 15 s. Figure 2b illustrates the scanning electron microscopy image of the micro-indentation on the foam wall.

The Vickers hardness is calculated using the formula $H_{v}\approx 0.1891\frac{F}{d^{2}}$, wherein *F* is the applied force and *d* is the arithmetic mean of the indentation diagonal. The relationship between the Vickers hardness number and yield strength is given by $H_{v}\approx 3\sigma_{ys}$ [2]. Experimental outcomes revealed that the foam core layer has an average Vickers hardness of 106.0 ± 8.74 Hv. Using this value, the yield strength of the core parent material is deduced to be 346.6 ± 28.58 MPa.

Several studies have employed Vickers micro-hardness tests on closed-cell aluminum foam manufactured via the powder metallurgy approach. For instance, IDRIS et al. [30], while investigating the ALULIGHT closed-cell aluminum foam (Al–Si10%–Mg1%) from Alulight International GmbH, identified the core parent material’s yield strength to be 179.3 ± 16.3 MPa. Similarly, McCullough et al. [31] found the yield strength of ALULIGHT (Al–Si0.6%–Mg1%) to be roughly 250 MPa, and for ALULIGHT (Al–Si10%–Mg1%), it was around 350 MPa.

### 2.3. Quasi-Static Compression Experiment

According to the Chinese standard GB/T 31930 [32], this research implemented quasi-static compression tests to determine the yield strength of the foam core. Initially, the upper and bottom panels of the AFS were eliminated using wire cutting. Following that, the foam core was segmented into samples boasting a height of 25 mm and a cross-sectional area measuring 20 mm × 20 mm. To eradicate any residual impurities within the pores from the fabrication process, the specimens underwent ultrasonic cleaning in both alcohol and deionized water, each lasting 5 min. The cleaned samples were placed in a forced-air drying oven for drying at ambient temperature. The density of the core layer of the aluminum foam sandwich panel in the three-point bending test ranges from 0.25 to 0.39 g/cm^3^. The samples used for compression experiments had a wider density range of 0.2 to 0.46 g/cm^3^. A wider density range allows for more accurate predictions of the relationship between yield strength and density. The number of samples in the compression experiments is 45. All tests were conducted at room temperature using the electronic universal testing machine AG-XPLUS (SHIMADZU, Kyoto, Japan), with a compression speed set at 2 mm/min.

Based on the stress-strain curve obtained from the compression test, the yield strength is identified as the compressive stress at its initial peak [23]. As a result, for sample densities spanning 0.20 to 0.46 g/cm^3^, the yield strength fluctuates between 1.41 and 7.92 MPa.

According to references [23,30], for closed-cell aluminum foam with an ideal structure, there exists a relationship between its yield strength and that of its parent material.

(1) σycσys=C1⋅(ρcρs)32+C2⋅(ρcρs)

In Formula (1), $\rho_{c}$ is the density of the foam core, and $\rho_{s}$ is the density of the parent material of the foam core. Coefficients *C*_1_ and *C*_2_ are associated with the geometric structure of the foam pores. Specifically, the formula’s initial term stems from the bending of the pore wall, and the second term is attributed to the surface yield when the pore wall undergoes stretching. According to the finite element analysis of TETRAKAIDECAHEDRAL closed-cell foam with flat faces presented in reference [30], the exponent of the first term in Formula (1) is set at 2. The values for *C*_1_ and *C*_2_ are established as 0.33 and 0.44, respectively.

**Figure 3:**
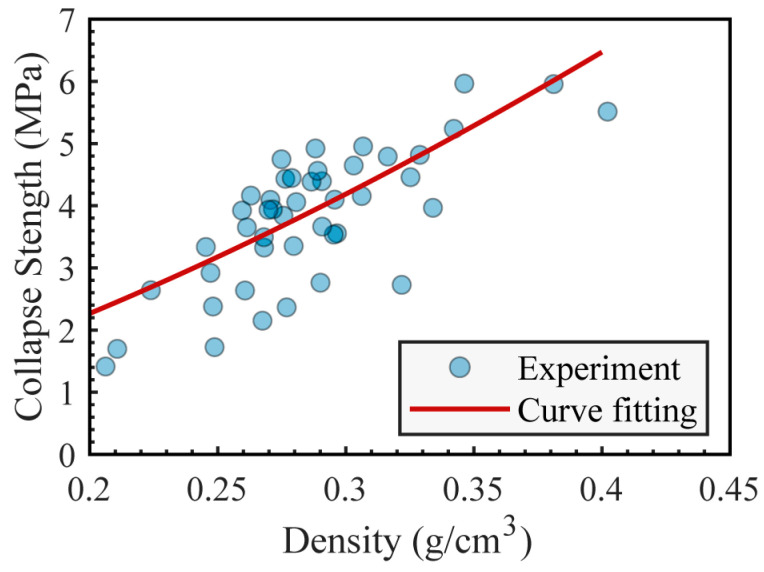
Curve fitting of aluminum foam’s yield strength in relation to its density.

From the compression test results, the yield strength $\sigma_{yc}$ of the aluminum foam was correlated with its density $\rho_{c}$. The relationship is illustrated in Figure 3, where $\sigma_{ys}$ equals 346.6 MPa and $\rho_{s}$ is 2.7 g/cm^3^. When applied to Formula (1), the exponent of the first term is determined as 1.513, with coefficients *C*_1_ and *C*_2_ being 0.3356 and approximately 0, respectively. Idris et al. [30] executed similar analyses for other aluminum foam varieties. Their results for ALPORAS aluminum foam suggest an exponent value of 6.95/2 for the first term in Equation (1), along with coefficient values of *C*_1_ = 0.44 and *C*_2_ = 0.1835. For the ALULIGHT aluminum foam, they deduced the exponent of the first term to be 4, with *C*_1_ = 0.01 and *C*_2_ = 0.0988. For metal structures with open cells, *C*_1_ = 1.5 and *C*_2_ = 0 [33].The structure [34] and composition [35] of the metal foam will affect its compression properties, so the fitting results will also be different.

Ideally, the shear strength of aluminum foam is approximately two-thirds of its uniaxial compressive strength [2,13], represented as $\tau_{yc}=2\sigma_{yc}/3$. However, this ratio can deviate based on the structural variances of the aluminum foam. For example, the ratio in reference [14] ranges between 0.4 and 0.5. For the aluminum foam samples prepared in this research, we sought to identify the shear to yield strength ratio. Employing the Chinese standard GBT-1455 [36], the shear strengths of aluminum foams across different densities were measured. They were then fitted with the yield strength calculated from Formula (1), resulting in $\tau_{yc}=C_{3}\sigma_{yc}$, where *C*_3_ has a value of 0.2909. By substituting this value into Formula (1), the relationship between core shear strength and core density can be obtained:

(2) $$\tau_{yc}=C_{1}C_{3}\sigma_{ys}{\left(\frac{\rho_{c}}{\rho_{s}}\right)}^{1.513}$$

### 2.4. Quasi-Static Three-Point Bending Tests

**Figure 4:**
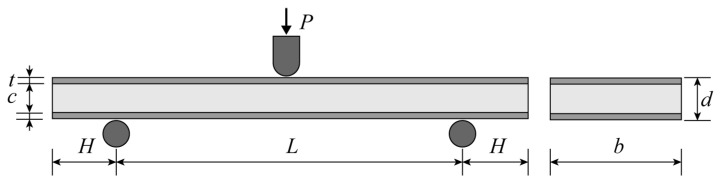
Schematic diagram of three-point bending of aluminum foam sandwich panels.

In accordance with the ASTM C393 [37] standard, we executed the three-point bending test at ambient temperature using the electronic universal testing machine AG-XPLUS, which boasts a maximum loading capacity of 10 KN and a consistent loading rate of 3 mm/min. The schematic of the three-point bending test is shown in Figure 4. A cylindrical hammer head with a radius of 5 mm applies a load *P* in the middle of the specimen. According to the ASTM C393 standard, the specimen width shall be not less than twice the total thickness and no more than one half the span length. The specimen length shall be equal to the support span length plus 50 mm. The span between the two support points is *L* (120 mm), and the distance by which the sandwich panel extends beyond the support points is *H* (25 mm). The thickness, width, panel thickness, and core thickness of the AFS are denoted as *d*, *b* (50 mm), *t*, and *c*, respectively.

During the experiment, Canon EOS R6 (Canon, Ota City, Japan) was used to take a photo every 10 s to record the deformation process of the samples. Each group of samples were tested three times. Table 3 delineates the specific parameters and outcomes of these tests, with a comprehensive analysis to follow in the subsequent section. It is pertinent to note that $\rho_{c}$ represents the foam core’s density. This is calculated by deducting the panel’s mass from the sandwich panel’s total mass, *m*, and subsequently dividing the value by the foam core’s volume. The panel’s mass is determined by multiplying its volume with the known density of aluminum, which is 2.7g/cm^3^.

## 3. Results and Discussion

### 3.1. Morphological Characterization of Foam Specimens

To characterize the foam core’s structure, one sample from each of the four groups was chosen. We used ImageJ to adjust the threshold of the aluminum foam image to obtain a binary image. At the same time, ImageJ was used to measure the number, diameter, and roundness of the pores. Importantly, to account for possible inaccuracies during binarization, pores smaller than 0.5 mm in diameter were excluded from the analysis.

**Figure 5:**
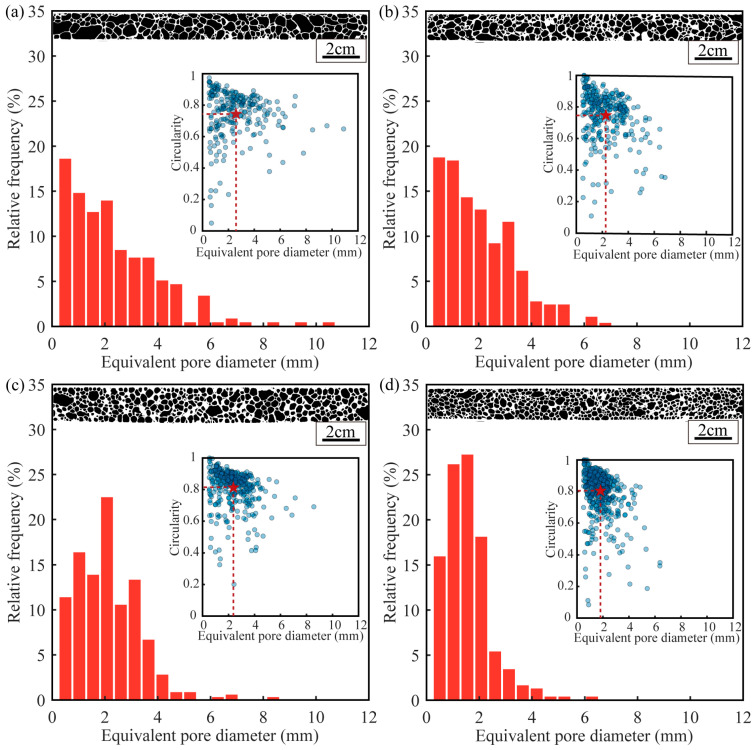
Distribution of equivalent pore diameters and cell circularity of aluminum foam sandwich panels with different densities: (**a**) sample A1, (**b**) sample B1, (**c**) sample C1, (**d**) sample D1.

Table 4 illustrates that as the density of the sample rises, the pore count similarly increases. Conversely, the standard deviation of the pore diameter decreases in tandem. Although samples A1 and B1 share a preform thickness of 4 mm and a foaming temperature of 650 °C, A1’s extended foaming time leads to greater pore merging. This results in A1 possessing larger pores than B1. Consequently, A1 has fewer pores overall, but their average diameter and standard deviation are notably larger. A comparison of Figure 5a,b reveals that A1 encompasses a higher number of large pores, with diameters surpassing 7 mm. For sandwich panels, where the core thickness is merely 14 mm, the existence of these sizable pores significantly influences its performance. Additionally, due to the height constraints of the mold, as the foaming time increases, the large pores are hindered in their upward growth. This causes the liquid core to flow sideways, resulting in a decrease in the density of the central foam core. For accurate three-point bending test sample preparation, it’s imperative to trim the edges of the foam aluminum sandwich panel, particularly the oxidized portions, preserving only the core’s central region. This is a prime factor behind A1’s lesser density compared to B1. The protracted foaming period for A1 directs more of the liquid core sideways, resulting in a sparser foam density at the center. Conversely, B1, with its condensed foaming time, experiences limited lateral liquid core flow, maintaining a denser central foam.

C1 and D1, despite sharing identical preform thickness and foaming temperature, exhibit varied results due to C1’s prolonged foaming time. As mentioned earlier, a longer foaming time leads to lower density, fewer pores, larger average pore diameter, and greater pore size standard deviation. C1 and D1 samples have more pores than A1 and B1. This discrepancy arises primarily because the greater preform thickness in C1 and D1 enhances hydrogen evolution, producing more pores during foaming. Additionally, the lower foaming temperature in C1 and D1 minimizes pore mixing, resulting in a smaller standard deviation of pores and heightened roundness. Also, the decreased foaming temperature heightens the viscosity of the molten aluminum in the core during C1 and D1’s foaming phase. This minimizes lateral aluminum liquid dispersion, ensuring a denser central foam core. As evident in Figure 5’s histogram, pore diameters in C1 and D1 predominantly lie between 1–3 mm. This indicates that the low foaming temperature and shorter foaming time have a significant impact on the core structure.

### 3.2. Deformation Modes

**Figure 6:**
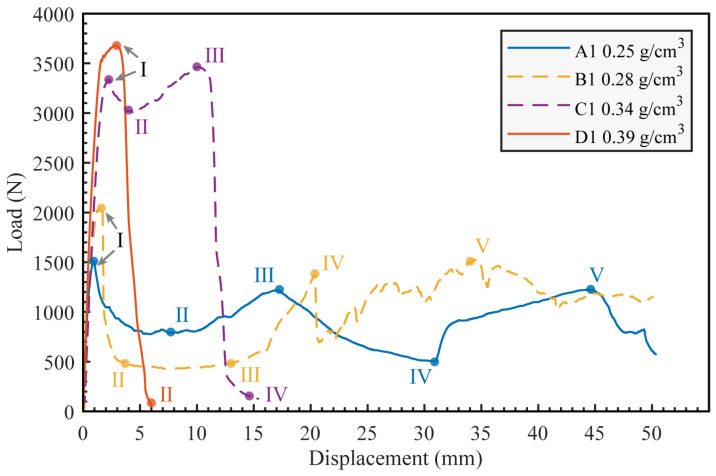
Load-displacement curves of aluminum foam sandwich panels with different densities.

The typical load-displacement curves of AFS with different densities are shown in Figure 6. In the initial small displacement phase, all samples exhibit elastic deformation. As density rises, so does the elastic modulus. Following this elastic phase, the curves transition into a nonlinear region, culminating in a peak load. Notably, a denser core results in a higher peak load. Beyond this peak, all samples display pores deformation and rupture, with the load decreasing as displacement augments. A denser foam core enhances the sandwich panel’s load-bearing capability. The maximum bending moment of the sandwich panel, denoted by *M*_max_, is deduced from the first peak load, *P*_max_, following the relation *M*_max_ = *P*_max_*L*/4 (as detailed in Table 3). In failure scenarios, AFS panels with denser cores can endure larger bending moments.

During our experiment, each of the four groups of samples exhibited distinct failure modes. Figure 6 presents the load-displacement curves specific to each mode. The four samples, A1 through D1, align with the following respective failure modes: (a) Mode I: indentation; (b) Mode II: core shear; (c) Mode III: a combination of indentation and face yield; (d) Mode IV: face yield.

**Figure 7:**
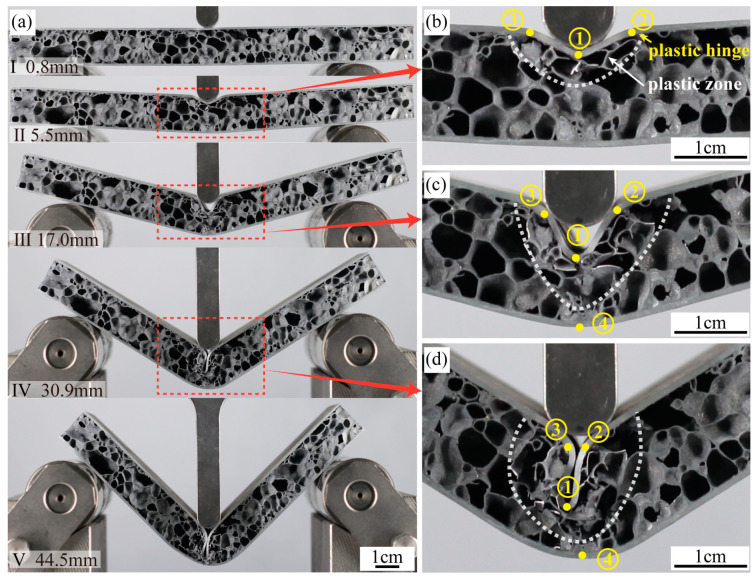
(**a**) The sequence of images shows the three-point bending test of A1, (**b**) displacement is 5.5 mm, (**c**) displacement is 17.0 mm, (**d**) displacement is 30.9 mm.

The failure mode of A1 is Mode I (indentation). The entire failure process is shown in Figure 7a. The evolving deformation patterns at different displacements, as illustrated in Figure 7a, correlate with A1’s load displacement in Figure 6. A1 attains its peak load at point I (d = 0.8 mm). At this point, there is no pronounced deformation discernible in the AFS. Transitioning from point I to II (d = 5.5 mm), there is a noticeable decline from the peak load. This decrement predominantly stems from the rupture of the foam pores, resulting in the compromised load-bearing efficacy of the sandwich panel. Delving deeper into Figure 7b, the deformation of the foam aluminum sandwich panel at point II is characterized by an indentation in the top panel coupled with the emergence of three plastic hinges. Contrarily, the bottom panel remains predominantly undistorted. Notably, the proximal region of the foam core adjacent to the top panel manifests a plastic deformation zone with evident pore ruptures, while the pores in the lower half remain unchanged.

Between points II to III (d = 17.0 mm), there is a gradual upswing in the load. This elevation can be chiefly attributed to the compaction of ruptured pores in the plastic zone identified at II. Concurrently, the bottom panel initiates substantial deformation, bolstering the sandwich panel’s load-bearing capacity. As shown in Figure 7c, the No.1 plastic hinge of the top panel at point III is detached from the hammerhead. This hinge intrudes deep into the core layer, compressing the foam core considerably, while the bottom panel generates the No.4 plastic hinge. Transitioning from III to IV (with a d = 30.9 mm), the plastic zone within the core layer continues its expansion, and a broader range of pores start to rupture, resulting in a gradual weakening of its load-bearing capability. As depicted in Figure 7d, by point IV, the No.2 and No.3 plastic hinges converge. The V-shaped deformation part of the upper panel is folded and fully inserted into the core layer. Transitioning from IV to V (d = 44.5 mm), the top and bottom panels’ folded sections further compact the ruptured pores in the plastic zone, perpetuating the load’s ascent. Throughout the deformation process, the foam core exhibits layered fracturing, and the top and bottom panels undergo staged deformation, leading to multiple fluctuations in the load.

**Figure 8:**
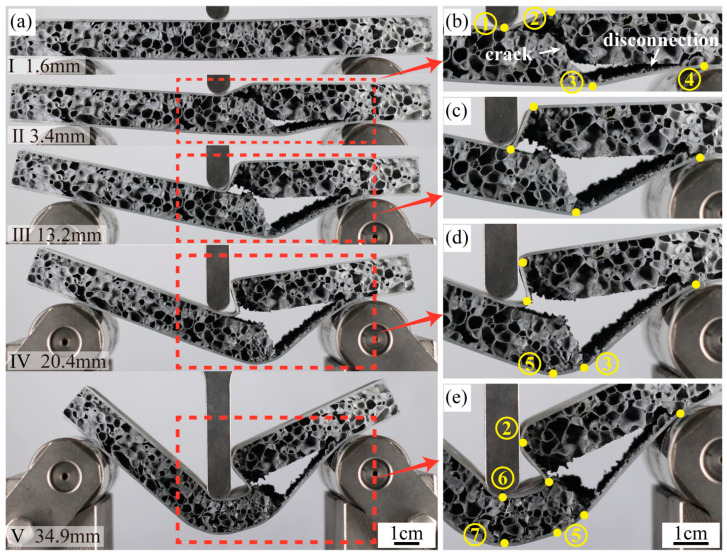
(**a**) The sequence of images shows the three-point bending test of B1, (**b**) displacement is 3.4 mm, (**c**) displacement is 13.2 mm, (**d**) displacement is 20.4 mm, (**e**) displacement is 34.9 mm.

B1’s failure mode is Mode II (core shear), and the deformation process is shown in Figure 8a. At point I, with a displacement of 1.6 mm, the load reaches its zenith, immediately followed by the shear failure evident within the core layer. As shown in Figure 8b, one can discern an oblique crack within the core layer at point II (d = 3.4 mm). The oblique crack traverses from the No.2 plastic hinge of the top panel to the No.3 plastic hinge of the bottom panel. Notably, both panels manifest two distinct plastic hinges. An apparent separation is observed between the core layer and these panels. This phase witnesses a precipitous load descent from 2055 N to 500 N, marking a reduction of approximately 76%, a decline significantly more pronounced than that of A1. The predominate rationale behind this core layer’s shear failure can be attributed to its amplified density, which subsequently bolsters the load-bearing prowess of the AFS. This shear failure is inaugurated once the load surpasses the core layer’s shear critical threshold.

During the transition from II to III (d = 13.2 mm), the load exhibits a marked stability. During this phase, both the top and bottom panels deform around the plastic hinges, and the foam cores remain undisturbed with neither significant deformation nor rupture. Notably, the top panel’s right side retains its horizontal orientation. During the transition from III to IV (d = 20.4 mm), the bottom panel forms a new No.5 plastic hinge. Due to the deformation, rupture, and further compression of the foam cores near No.3 and No.5 plastic hinges, the load shows an upward trend. From IV to the end, both panels exhibit new plastic hinges, labeled No.6 and No.7, situated directly beneath the hammer. The foam cores undergo sustained rupture and compression, primarily beneath the hammer and the No.2 plastic hinge. As a consequence, the load fluctuates at a stable horizontal level.

**Figure 9:**
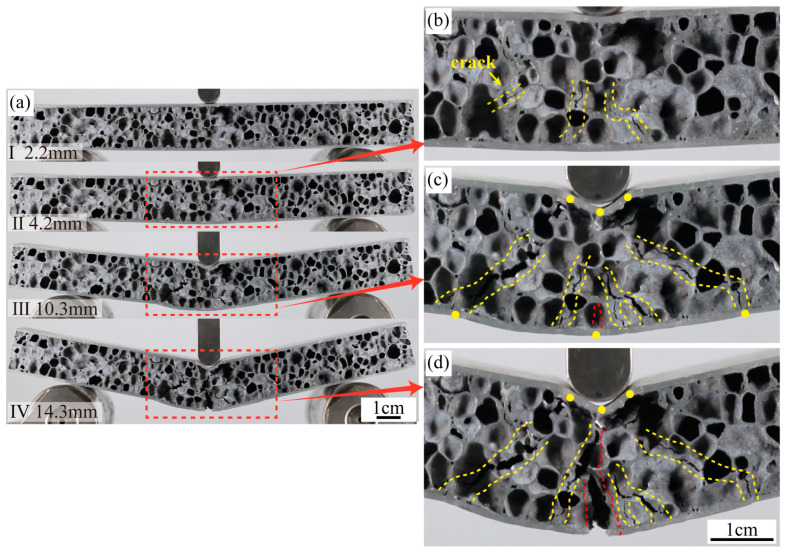
(**a**) The sequence of images shows the three-point bending test of C1, (**b**) displacement is 4.2 mm, (**c**) displacement is 10.3 mm, (**d**) displacement is 14.3 mm.

The failure mode of C1 is Mode III, with its deformation process illustrated in Figure 9a. The sample reaches its peak load at point I (d = 2.2 mm). As the displacement increases, several cracks appear in the foam core directly beneath the hammer, as shown in Figure 9b. Given the heightened foam core density, the core’s ability to bear the load intensifies, achieving a peak load of 3336 N. This value is 2.22 times greater than A1’s peak load. Thus, the failure mode of C1 significantly differs from that of A1. Cracks in A1 initially appear in the upper half of the core and gradually extend to the lower half, while in C1, cracks emerge from the onset in the lower half of the core. During the phase from II to III (d = 10.3 mm), the area of foam core rupture expands, accompanied by multiple cracks. Due to the intensified deformation of the upper and bottom panels, the load increases, reaching a new peak. As shown in Figure 9c, the upper panel is indented and forms three plastic hinges. The pores directly under the hammer also rupture, which is similar to the characteristics of Mode I. Additionally, several oblique cracks appear, along with minor plastic hinges at the bottom panel, resembling the features of Mode II. From III to IV (d = 14.3 mm), the bottom panel succumbs to deformation and subsequently fractures, leading to a decline in the sandwich panel’s load-bearing ability. During the phase from I to III, despite several ruptures in the foam core, the load did not drop significantly and consistently remained above 3000 N. Such a sustained load surpasses the bearing threshold of the bottom panel, culminating in its fracture.

**Figure 10:**
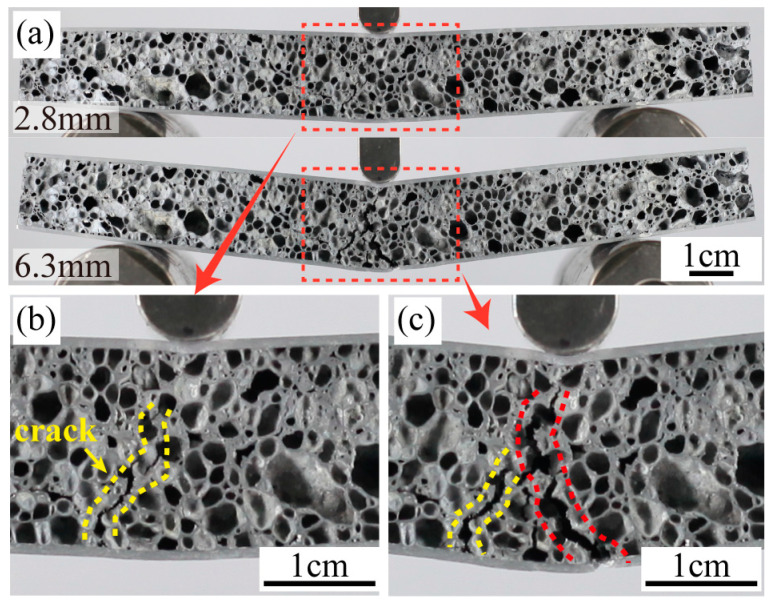
(**a**) The sequence of images shows the three-point bending test of D1, (**b**) displacement is 2.8 mm, (**c**) displacement is 6.3 mm.

D1’s failure mode is Mode IV, as illustrated in Figure 10. The peak load is attained at point I (d = 2.8 mm). At this point, cracks become evident in the foam core’s lower section, as highlighted in Figure 10b. This is similar to the failure process of C1. However, given that D1’s core density surpasses that of C1, its peak load registers a commensurate escalation. This heightened load implies that D1’s central foam core refrains from manifesting further ruptures but rather induces a direct fracture of the bottom panel, as shown in Figure 10c. It’s imperative to note that while amplifying the core’s density can enhance the sandwich panel’s load-bearing capability, it may precipitate a premature fracture of the thinner panel. Such an occurrence can undermine the intended efficacy and utility of the AFS in real-world applications.

### 3.3. Failure Load Prediction and Failure Mechanism Map

The failure mode of AFS in the three-point bending test is influenced by variations in core density. Table 3 lists the failure modes for each sample group. Group A has the lowest core density, with a failure mode of Mode I (indentation) and an initial failure load of 1561.83 ± 56.32 N. In this mode, the upper panel undergoes indentation, leading to cracks in the upper half of the foam core. Conversely, the bottom panel remains undeformed. The failure load is calculated using Formula (3) [25]:

(3) $$P_{in}=2bt\sqrt{\sigma_{yf}\sigma_{yc}}$$

The yield strength $\sigma_{yf}$ of the panel, obtained through a tensile test, is 78 MPa. The yield strength $\sigma_{yc}$ of the core is calculated using Formula (1), resulting in a relationship between failure load and core density:

(4) $$P_{in}=2bt\sqrt{C_{1}\sigma_{yf}\sigma_{ys}{\left(\frac{\rho_{c}}{\rho_{s}}\right)}^{1.513}}$$

where *C*_1_ = 0.3356, and the yield strength $\sigma_{ys}$ of the core parent material is 346.6 MPa.

The failure mode for Group B is Mode II, with an initial failure load of 1848.56 ± 153.38 N. Given its higher density compared to Group A, Group B inherently possesses superior load-bearing capabilities. Due to the excessive failure load, the foam core experiences shear failure. At the beginning of failure, the core exhibits oblique cracks and detaches from the panel. Such separation acts as a stress-relief mechanism, causing damage to manifest only on one side of the core. This initial failure mode aligns with the Mode IIB shear failure mode mentioned in reference [13], and the formula to calculate the failure load is

(5) $$P_{cs}=2\sigma_{yf}\frac{bt^{2}}{L}+2bc(1+\frac{H}{L})\tau_{yc}$$

By substituting into Equation (2), we can derive the relationship between shear failure and core density:

(6) $$P_{cs}=2\sigma_{yf}\frac{bt^{2}}{L}+2bcC_{1}C_{3}\sigma_{ys}(1+\frac{H}{L}){\left(\frac{\rho_{c}}{\rho_{s}}\right)}^{1.513}$$

The initial failure loads of Group C and Group D are 3273.15 ± 78.77 N and 3635.54 ± 45.14 N, respectively. Since these peak loads surpassed the endurance limits of their bottom panels, the panels yielded and eventually fractured. Among the two, Group C, having a lower density and consequently a larger average pore diameter, demonstrated a complex failure mode. The failure modes for C1 and C2 are Mode III. At the initial peak load, the upper panel of these samples undergoes indentation, while the core displays oblique cracks, as shown in Figure 9c. The sample completely failed upon reaching the second peak load. At this moment, the bottom panel exhibited yielding and fracturing [14], and cracks emerged throughout the entire core layer. When the D group samples failed, there was no indentation in the upper panel, with only a few oblique cracks appearing in the core layer. Both Group C and Group D showed yielding and fracturing of the bottom panel upon reaching the peak load, with new cracks forming in the center of the core layer. For these thin-panel samples, the load induced by the core yielding [23] cannot be ignored. The formula for calculating the failure load at this moment is

(7) $$P_{fy+cy}=\frac{4bt(c+t)}{L}\sigma_{yf}+\sigma_{yc}\frac{bc^{2}}{L}$$

Substituting into Formula (1) gives the relationship between the failure load and density:

(8) $$P_{fy+\mathrm{cy}}=\frac{4bt(c+t)}{L}\sigma_{yf}+C_{1}\frac{bc^{2}}{L}\sigma_{ys}{\left(\frac{\rho_{c}}{\rho_{s}}\right)}^{1.513}$$

**Figure 11:**
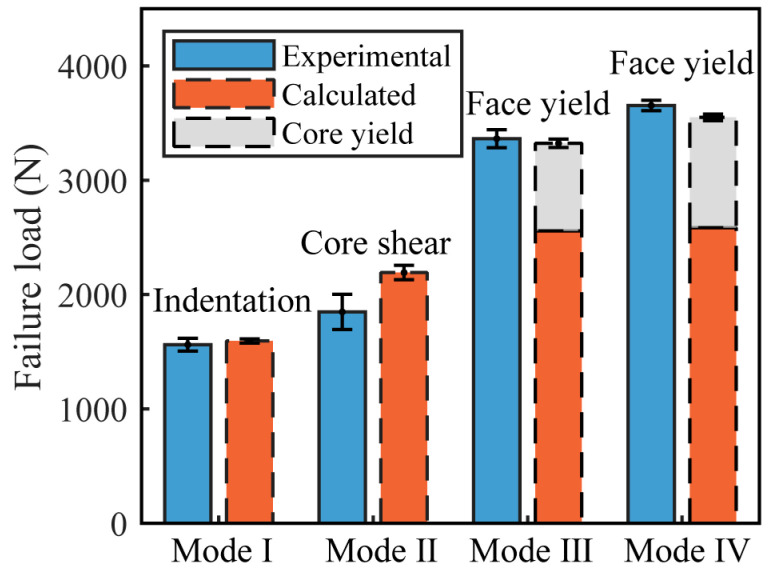
Comparison between the experimental and predicted failure loads under different failure modes.

**Figure 12:**
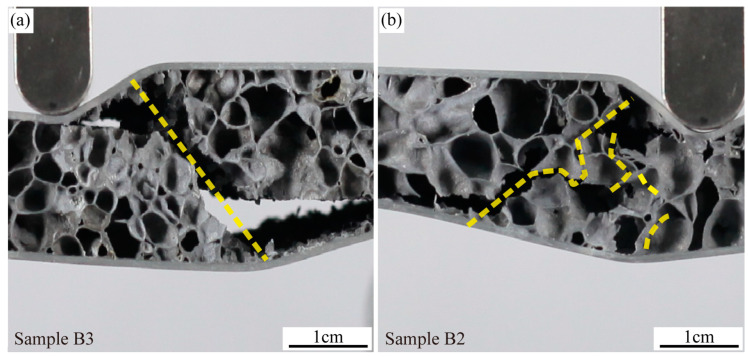
Sample B3 (**a**) and sample B2 (**b**) failed by core shear.

The comparison of the experimental values and theoretical values of the failure loads for each group is shown in Figure 11. The experimental outcomes match well with the theoretical predictions across all four failure modes. As shown in Table 3, the core density from A to D increases in sequence. The average core thickness for groups C and D is 18.50 mm, while for groups A and B, it is 14.26 mm. As the core density and thickness increase, the aluminum foam sandwich panel’s load-bearing capability is enhanced, leading to an increase in the failure load. For instance, the failure load of the D group panel yielding is 2.33 times that of the A group panel indentation. It is noteworthy that the peak load estimations for core shear failure show a larger deviation than those for other failure modes. This discrepancy is due to the uneven distribution of cell sizes within the sample. Although the samples in Group B have the same density, the cells beneath the hammerhead in sample B3 are larger than those in B2, as illustrated in Figure 12. During core shear failure, the smaller cells in B3 are less likely to be crushed, resulting in only a single oblique crack. In contrast, sample B2 exhibits both an oblique crack and cell rupture. Consequently, the peak load of B3 is greater than that of B2. The considerable variability in the oblique crack patterns during core shear failure contributes to the observed inaccuracies in predicting peak loads.

**Figure 13:**
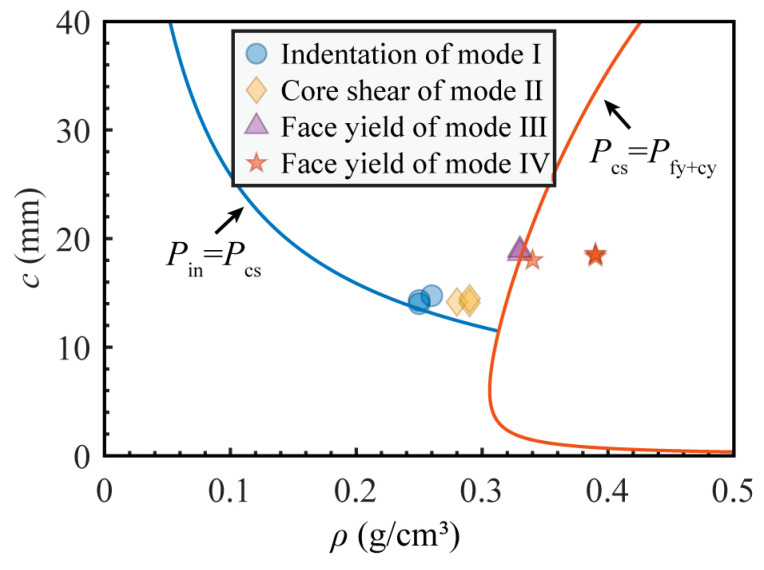
Figure map of three-point bending tests of aluminum foam sandwich panels with varying core densities and thicknesses.

Figure 13 illustrates the influence of variations in core layer density and thickness on the failure mode. The critical line for the indentation and core shear modes is derived from Formulas (4) and (6), while the critical line between core shear and face yield (along with core yield) is derived from Formulas (6) and (8). From Figure 13, it can be observed that all the data points of Group D on the right side of the boundary line exhibit the failure mode of face yield. Group C is close to the dividing line; the variability in pore size has a significant impact on the failure modes. In Group C, when the face yields, oblique cracks may appear in the core layer and the upper panel may be dented. Therefore, the failure mode of C3 is different from that of other Group C samples. This critical line is very crucial. To prevent the bottom panel from fracturing, the density and thickness of the form cores should not exceed this critical value during the production process. Groups A and B are not distinctly separated by the critical line, this is because both groups are close to the critical line and there is a certain prediction error; similar situations have also appeared in other studies [14,16]. However, when there is a significant difference in the density and thickness, the critical line between indentation and core shear still has reference value.

### 3.4. Energy Absorption

**Figure 14:**
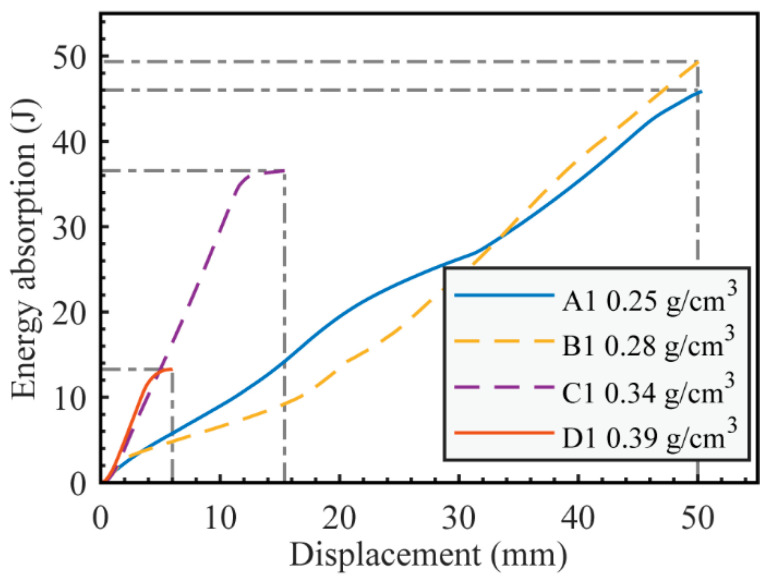
Energy absorption curves for specific samples.

As shown in Figure 14, energy absorption curves for different failure modes were derived by integrating the load-displacement curve presented in Figure 6. For the face yield mode, its pronounced initial failure load leads to a superior early energy absorption efficiency, surpassing that of indentation and core shear. However, its overall energy absorption capability trails behind. This is because the fracture of the bottom panel causes it to fail prematurely, impeding its sustained load-bearing ability. C1 completely fails at about 15 mm displacement, while D1 completely fails at a displacement of 5 mm. In contrast, A1 (indentation) and B1 (core shear) still have a certain load-bearing capacity even at a 50 mm displacement.

**Figure 15:**
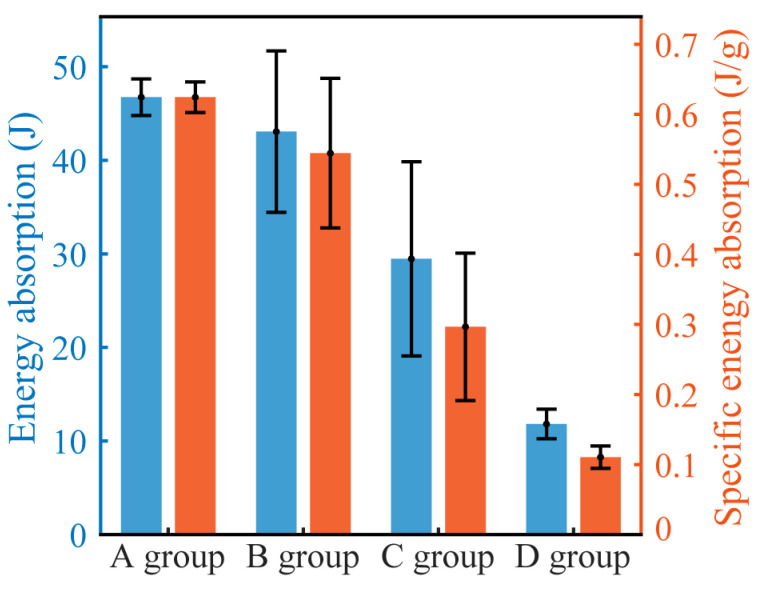
Comparison of energy absorption and specific energy absorption among the groups.

Figure 15 offers a comparative analysis between the average energy absorption values and the average specific energy absorption values of the four sample groups. The specific energy absorption value is obtained by dividing the energy absorption value by the total mass *m* of the sample. From Figure 15, it is evident that as the density of the samples increases, the average energy absorption value gradually decreases. Notably, groups A (indentation) and D (face yield) exhibit relatively minor standard deviations, suggesting a uniformity in energy absorption values among individual samples within these groups. This is because under these two failure modes, the rupture of the foam core pores is mainly confined to a small central area, hence there is minimal variation between different samples. The energy absorption standard deviation for Group B (core shear) is relatively large, because when the core shear failure occurs, there is significant variation in the slanted cracks of the foam core, resulting in notably different energy absorption values. Group C is located at the boundary line (as shown in Figure 13), and, therefore, two failure modes exist, resulting in a larger standard deviation for energy absorption values. Because the mass of groups C and D is relatively larger, their specific energy absorption is lower compared to groups A and B.

## 4. Conclusions

By optimizing process parameters, this research achieved the preparation of AFS characterized by varied core densities and thicknesses. The three-point bending tests executed on these panels provided insights into how core density impacts their failure mode, load-bearing capability, and energy absorption efficiency. The conclusions are as follows:

1. The higher the foaming temperature and the longer the foaming time, the lower the core density of the AFS, the fewer the number of foam cores, and the larger the average core diameter.
2. As the core density increases, the load-bearing capacity of the AFS is significantly enhanced, resulting in a marked increase in the bending strength and the threshold for failure load.
3. AFS panels with different core densities exhibit various failure modes. At a core density of approximately 0.26 g/cm^3^, the top panel undergoes indentation failure, accompanied by layer-by-layer rupture of foam cores.
4. When the core density reaches about 0.29 g/cm^3^, the AFS’s failure mode is core shear. When the core density exceeds 0.33 g/cm^3^, the peak load surpasses the bearing threshold of the bottom panel, resulting in its yield and fracture.
5. The initial failure loads for various failure modes were projected theoretically, and it was found that the calculated results align well with the experimental values. Based on these theoretical predictions, a failure mode diagram was designed, serving as a guide for the production of thin-panel aluminum foam sandwich panels.
6. When the core density of AFS is greater than 0.33 g/cm^3^, it exhibits an elevated initial energy absorption efficiency. However, this also leads to premature failure. In the context of large deformations, AFS with a core density of less than 0.29 g/cm^3^ demonstrate superior energy absorption efficiency.

## Figures and Tables

**Table 1:** Elemental composition of mixed powders [10].

Composition	Range Size (μm)	Purity (%)	Content
Al	<45	99.7	85%
Si	<38	99.5	6%
Mg	<75	99.9	4%
Cu	<38	99.9	4%
TiH_2_	<45	99.7	1%

**Table 2:** Parameters for sample fabrication.

Label	Panel Size (mm)	Cavity Height (mm)	Powder Weight (kg)	Preform Thickness (mm)	Foaming Temperature (°C)	Foaming Time (min)
A	500 × 350 × 4	15	4.2	4	650	16
B	500 × 350 × 4	15	4.5	4	650	12
C	500 × 350 × 4	20	5.6	6	620	16
D	500 × 350 × 4	20	6.0	6	620	12

**Table 3:** Dimensions and results of the three-point bending test of the sandwich panels.

Label	*c* (mm)	*t* (mm)	*m* (g)	*ρ*_c_ (g/cm^3^)	*P*_max_ (N)	*P*_pre_ (N)	*M*_max_ (Nm)	Failure Mode
A1	14.00	0.98	75.14	0.25	1510.39	1619.70	45.31	IN
A2	14.74	0.95	75.48	0.26	1534.87	1584.99	46.05	IN
A3	14.10	0.96	73.93	0.25	1640.22	1577.86	46.21	IN
B1	14.16	0.99	79.13	0.28	1701.34	2109.27	51.04	CS
B2	14.10	0.96	78.46	0.29	1784.21	2207.87	53.53	CS
B3	14.47	0.93	79.48	0.29	2060.13	2260.55	61.80	CS
C1	18.02	1.02	98.81	0.34	3465.32	3270.89	100.12	IN + FY
C2	18.65	1.01	99.65	0.33	3354.12	3345.32	103.62	IN + FY
C3	18.94	0.99	100.00	0.33	3273.15	3323.04	98.19	FY
D1	18.50	1.02	105.41	0.39	3679.15	3556.26	110.37	FY
D2	18.31	1.03	107.33	0.39	3573.35	3511.28	107.20	FY
D3	18.61	1.02	108.61	0.39	3654.12	3582.39	109.62	FY

Note: The failure modes presented in the table are abbreviations. ‘IN’—Indentation; ‘CS’—Core shear; ‘FY’—Face yield.

**Table 4:** Statistical results of the cell parameters of foams.

Label	Total Count of Pores	Equivalent Pore Diameter (mm)	Standard Deviation of Equivalent Pore Diameter	Average Circularity
A1	237	2.59	1.76	0.74
B1	294	2.28	1.34	0.75
C1	361	2.37	1.17	0.81
D1	559	1.81	0.87	0.80

## Data Availability

The processed data required to reproduce these findings can not be shared at this time, as the data also form part of an ongoing study.
